# Effects of Radio Frequency Tempering on the Texture of Frozen Tilapia Fillets

**DOI:** 10.3390/foods10112663

**Published:** 2021-11-02

**Authors:** Jiwei Jiang, Fen Zhou, Caining Xian, Yuyao Shi, Xichang Wang

**Affiliations:** 1 College of Food Science and Technology, Shanghai Ocean University, Shanghai 201306, China; m180300676@st.shou.edu.cn (J.J.); d170202044@st.shou.edu.cn (F.Z.); m190300801@st.shou.edu.cn (C.X.); m190310917@st.shou.edu.cn (Y.S.); 2 Shanghai Aquatic Product Processing and Storage Engineering Technology Research Center, Shanghai 201306, China; 3 Laboratory of Quality and Safety Risk Assessment of Aquatic Products Storage and Preservation, Ministry of Agriculture, Shanghai 201306, China

**Keywords:** tempered fillets, electrode gap, springiness, cohesiveness, resilience, hardness

## Abstract

Radio frequency (RF) tempering has been proposed as a new alternative method for tempering frozen products because of its advantages of rapid and volumetric heating. In this study, the texture of RF-tempered frozen tilapia fillets was determined under different RF conditions, the effects of related factors on the texture were analyzed, and the mechanisms by which RF tempering affected the texture of the tempered fillets were evaluated. The results show that the springiness (from 0.84 mm to 0.79 mm), cohesiveness (from 0.64 mm to 0.57 mm), and resilience (from 0.33 mm to 0.25 mm) decreased as the electrode gap was increased and the power remained at 600 W, while the shear force increased as the power was increased for the 12 cm electrode gap (from 15.18 N to 16.98 N), and the myofibril fragmentation index (MFI) values were markedly higher at 600 W than at 300 W or 900 W (*p* < 0.05). In addition, the tempering uniformity had a positive effect on hardness and chewiness. The statistical analysis showed that the texture after RF tempering under different RF conditions correlated relatively strongly with the free water content, cooking loss, and migration of bound water to immobilized water. The decrease in free water and bound water migration to immobilized water resulted in a significant increase in cohesiveness and resilience.

## 1. Introduction

“Tilapia” is the name given to several genera and species of fish in the family Cichlidae, which have been proven to be among the most important food fish in the world and are popular because of their high protein content [1]. Tilapia are generally processed into frozen fillets for transportation and sales. Fish are among the most highly perishable food products [2], and further quality deterioration in freshness occurs during fishing, storage, transport, and processing due to the presence of rich endogenous enzymes and microorganisms, and therefore, freezing is a common method of fish preservation [3]. For further processing, frozen tilapia fillets need to be defrosted, which is also a key operation for meeting the needs of consumers. However, traditional tempering methods affect the quality, water and juice loss, texture, and organizational structure of fillets by causing varying degrees of damage due to changes in physical properties, chemical reactions, and microorganisms [4], which lead to increased resource consumption due to longer tempering times. Air tempering has disadvantages, such as a slow tempering rate and greater susceptibility to microbial contamination and lipid oxidation. Tempering with running tap water easily causes secondary pollution, considerable juice loss, and tissue collapse. Low-temperature–high-humidity tempering needs a larger processing site, and the investment cost is large [5,6].

New tempering technologies, such as high-voltage electrostatic field (HVEF) tempering, radio frequency (RF) tempering, ohmic tempering, microwave tempering, pressure-assisted tempering, and acoustic tempering, are under consideration by those people working to solve the problems with the tempering process mentioned above [7,8,9,10]. The emerging tempering technologies have advantages such as shorter tempering times, low energy consumption, high energy utilization rates, effective inhibition of the growth of microorganisms and lipid oxidation during the tempering process, reduced production of spoilage substances, improved freshness, and extended storage time. Electrical treatments play important roles as alternative methods for processing foods with minimal damage. According to Yang et al. [11], RF tempering technology has led to shorter tempering times and lower energy consumption. RF technology is particularly suitable for heating samples compared with other novel tempering technologies because of its low frequency and deep penetration depth [12]. The volumetric nature of RF tempering shortens the processing time and increases the heating rate, which can improve the sample tissue characteristics in ways that are different from conventional tempering methods. The change in temperature in different parts (tempering uniformity) of a sample and the thermal and non-thermal effects during the RF tempering process can affect the microstructure and macrostructure of the product and cause a variety of phenomena (including water migration and texture changes), depending on the tempering conditions [13].

Water exists inside and in the interstices of myofibrils in muscle tissue and forms compartments, which can complicate the internal change process during tempering, affect the steady state of the complex meat system, and, in turn, affect the texture after tempering [14]. According to some reports, the loss of water retention capacity can lead to the destruction of muscle fiber structure, and the shrinkage of muscles during processing also has a relationship with the distribution of water [15,16]. Therefore, tempering uniformity, water loss, and moisture migration may also affect the final texture. Bedane et al. [13] reported that increasing the electrode gap can improve the hardness of chicken after RF tempering. Farag et al. [17] found that RF tempering reduced the loss of micronutrients and water in beef during the tempering process compared with conventional methods. Zhang et al. [18] demonstrated that a higher L* value was achieved in tilapia fillets tempered by RF. Although the researchers have confirmed that RF tempering can improve some qualities compared with conventional tempering [13,17,18,19], there was less research on texture, and the mechanism has also not been studied and remains to be further clarified. Therefore, the purposes of this study were to analyze the texture of frozen tilapia fillets under different RF tempering conditions (power and electrode gaps) and to explore the mechanism of the changes in texture for different RF treatments based on tempering uniformity, water loss, and moisture migration, which will provide a useful reference for exploring quality improvements in RF-tempered products.

## 2. Materials and Methods

### 2.1. Sample Preparation, Radio Frequency Tempering, and Temperature Measurement

**Figure 1:**
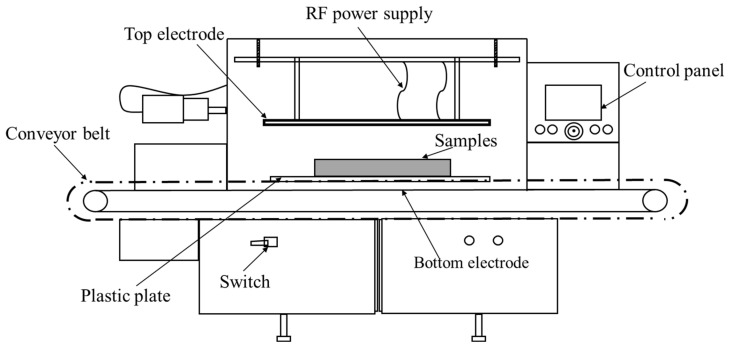
Schematic diagram of radio frequency (RF)-tempering experimental device.

Vacuum-packed frozen tilapia fillets (the weight of each fillet was 155 ± 10 g and the length was 18.8 ± 1.0 cm) were purchased from TongWei Aquatic Products Company (Hainan, China) and stored at −19 ± 1.0 °C. A piece of fillet was tempered in each experiment, which was repeated in at least 3 batches; at least 200 pieces of fillet in total were used in this study. A 50-ohm RF heater (27.12 MHz) (Labotron12, Sairem, Lyon, France) (Figure 1) was used for the RF treatments. Three electrode gaps of parallel-plate RF equipment were selected (10, 12, and 14 cm), and the three power inputs were 300, 600, and 900 W.

In the stationary experiments, the fish block was placed at the horizontal center of the electrodes. For recording changes in the temperatures of the samples during RF tempering, a 6-channel signal conditioner was used and fiber optic probes (HQ-FTSD120, HeQi Technologies Inc., Xi’an, China) were inserted into the center of its thickest part for each sample. The tempering process was terminated when the temperature reached −4 °C. In the present investigation, the endpoint temperature for tempering was set to approximately −4 °C, because this temperature is typically used in the meat and fish industry for the handling and manipulation of tempered meat blocks [20]. The surface temperature was measured after tempering using an infrared thermal image system (FLIR A655SC, Portland, OR, USA), and surface temperature distribution analysis was carried out using an image analysis system (FLIR Tools). The sample was positioned to ensure that the camera recorded an image of the whole upper surface.

### 2.2. Texture Determination

#### 2.2.1. Texture Profile Analysis (TPA)

The texture profile analysis test was performed using a model TA-XT2i texture analyzer (Stable Micro Systems, Surrey, UK) with a 5 kg compression load and 20% deformation. The pre-test speed was 1 mm/s, the test speed was 1 mm/s, and the post-test speed was 10 mm/s. The textural parameters of hardness (the hardness generally represents the force exerted by the molars to bite the sample for the first time), springiness (the springiness refers to the ability of the sample to return to its original state after extrusion), chewiness (the chewiness indicates the resistance of the sample to chewing, which is the product of hardness, springiness, and cohesiveness), and cohesiveness (the cohesiveness reflects the internal binding ability of the sample to resist damage and maintain its integrity) were derived from a force–time curve [21].

#### 2.2.2. Warner–Bratzler Shear Test

The fish samples were trimmed into 3 cm × 2 cm × 1.5 cm (length × width × height) pieces. Measurements were taken immediately, and the shear blade was set vertical to the direction of the meat fibers using a Warner–Bratzler shear force blade attached to a model TA-XT2i texture analyzer (Stable Micro Systems, Surrey, UK) [22]. The pre-test speed was 10 mm/s, the test speed was 2 mm/s, and the post-test speed was 10 mm/s. The distance and trigger force were 30 mm and 50 g, respectively.

#### 2.2.3. Measurement of the Myofibril Fragmentation Index (MFI)

The MFI was determined according to the method of Zou et al. [22]: 3.00 g of the minced fish sample was accurately weighed and homogenized for 1 min in 30 mL of phosphate buffer (100 mmol/L KCl, 7 mmol/L KH_2_PO_4_, 18 mmol/L K_2_HPO_4_, 1 mmol/L EDTA, and 1 mmol/L MgCl_2_, at pH 7.0, stored at 4 °C). Then, the sample was centrifuged at 10,614× *g* for 30 min at 4 °C, and the precipitate was resuspended in 30 mL of phosphate buffer and centrifuged again. The precipitate was dissolved in 15 mL of extraction solution and then filtered with a single layer of gauze to remove connective tissue and cell debris. The filtrate was the myofibril extract. The protein concentration of the myofibril extract was diluted to 0.5 mg/mL and measured spectrophotometrically at 540 nm, and the results were calculated according to Equation (1):

MFI = A540 nm × 200
(1)

### 2.3. Determination of Drip Loss and Cooking Loss

#### 2.3.1. Drip Loss

Tilapia fillets (frozen fillets and fillets that had been tempered and treated to remove surface water) were accurately weighed according to the method of Choi et al. [19], and the drip loss was calculated according to Equation (2):

(2) $$\mathrm{Drip}\mathrm{loss}(\%)=\frac{M_{0}-M_{t}}{M_{0}}\times 100$$

M_0_ is the weight of frozen tilapia fillets before tempering (g) and M_t_ is the weight of fillets after they were tempered and treated to remove the surface water (g).

#### 2.3.2. Cooking Loss

The cooking loss was determined as described by Li et al. [3]. Based on the weight of the meat before and after cooking, the cooking loss of the sample was calculated according to Equation (3):

(3) $$\mathrm{Cooking}\mathrm{loss}(\%)=\frac{M_{e}-M_{f}}{M_{e}}\times 100$$

M_e_ is the the initial weight of the sample before cooking and M_f_ is the final weight of the sample after cooking

### 2.4. Moisture Distribution and Migration

The distribution and migration of moisture in the tilapia fillet samples were analyzed by the relaxation time (T_2_) of the GCMPs gel determined using a Niumag Benchtop Pulsed NMR Analyser PQ001 (Niumag Electric Corporation, Shanghai, China). The main parameters were: P_1_ = 18 μs, P_2_ = 36 μs, NS = 6 and NECH = 3000.

### 2.5. Statistical Analysis

All the experiments were performed in triplicate. The data were reported as mean ± standard deviation (SD) for each triplicate treatment. Analysis of significant differences (*p* < 0.05) was performed using one-way analysis of variance (ANOVA) calculated by SPSS 20.0 software (SPSS Inc., Chicago, IL, USA). Origin 2018, Origin 2021b and Microsoft PowerPoint-2016 were used to plot and combine figures.

## 3. Results and Discussion

### 3.1. Tempering Curve

**Figure 2:**
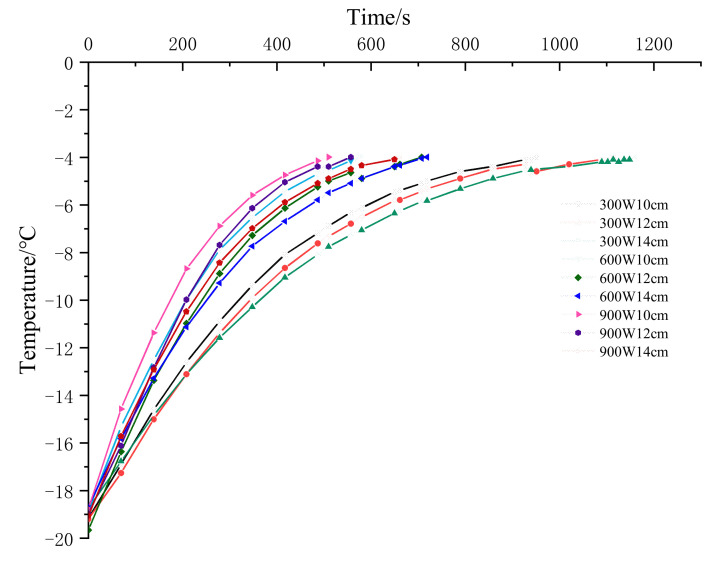
Internal center temperature curve of RF tempering treatment group with different electrode gaps and powers.

Figure 2 shows the temperature variation in the tempered tilapia fillets subjected to different methods. The tempering times of the 10 cm and 12 cm samples remained lower than those of the 14 cm group. The tempering times of the 300 W samples significantly exceeded those of the 600 W and 900 W groups. The tempering times of 511 s and 1162 s for 900 W-10 cm and 300 W-14 cm were ensured, and were the shortest and longest, respectively. Increasing the power or reducing the electrode gap increased the tempering rate.

### 3.2. Texture

#### 3.2.1. Texture Profile Analysis (TPA)

The textures of the tilapia fillet samples after RF treatment are presented in Table 1. The springiness, cohesiveness, and resilience decreased as the power increased for the 10 cm electrode gap. There was a decreasing trend in the textural characteristics of springiness, cohesiveness, and resilience when the power was retained at 600 W and the electrode gap was increased. These results agree with those reported by Bedane [13], who found that the springiness and chewiness of melted chicken breasts did not increase significantly with increases in the electrode gap during RF tempering. The hardness values first increased and then decreased after treatment with increasing power when the electrode gap remained unchanged.

This result was related to the shorter exposure time (to the electric field and air) for the 600 W than 300 W samples. Small ice crystals were not completely melted, and there was less cell loss that, together with the relatively intact muscle tissue and slower protein denaturation, made the tissue not loose. However, at 900 W, the energy accumulation increased, and the temperature distribution was uneven due to the strong electric field deflection, which caused the tissue to collapse with increased juice loss from the fillet edge [23]. Therefore, all of these factors led to the aforementioned trends. The hardness gradually increased with the increasing electrode gap at 900 W, possibly because the electric field was gradually deflected with the increasing electrode gap, which reduced the electric field concentration. The temperature increased slowly per unit time, and the ice crystals melted more slowly, so the tissue was less damaged, and the tissue firmness of the fillets was better maintained [13]. For the 10 cm and 12 cm electrode gaps, the chewiness and hardness of the fillets tempered at different powers showed the same trend, and the chewiness of the fillets tempered in the 600 W group was better than that of fillets subjected to other powers.

#### 3.2.2. Warner–Bratzler Shear Test

There is an inverse relationship between the shear force exerted on muscle and tenderness. The shear force upon the tilapia fillets tempered with a 10 cm electrode gap was significantly lower than those of the 12 cm and 14 cm groups at the same power (*p* < 0.05), as shown in Table 1. These results are related to the increases in thermal conductivity and the loss of some soluble substances with the decrease in electrode gap. The findings show that the shear force increased as the power increased under the 12 cm electrode gap. During heating, different muscle proteins denature and cause structural changes, such as the destruction of cell membranes, the transversal and longitudinal shrinkage of meat fibers, aggregation, the gel formation of sarcoplasmic proteins, and the shrinkage and solubilization of connective tissue [16]. However, the tempering time decreases as the power increases, and there is less damage to mitochondria, sarcoplasmic reticulum, and lysosome membrane and less release of salt-soluble proteins and tenderizing enzymes. Therefore, a lower tempering time reduces the enrichment of salt-soluble proteins on the surface of the meat, and plays a role in reducing meat tenderness [22].

#### 3.2.3. Myofibril Fragmentation Index (MFI)

**Figure 3:**
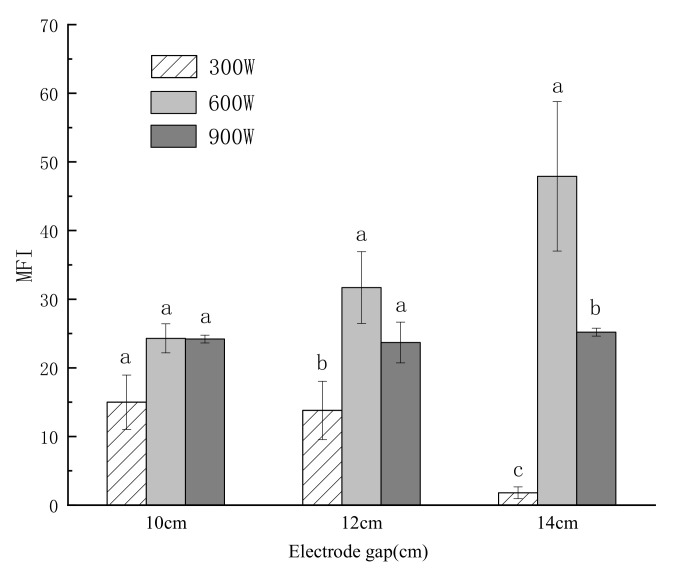
Effects of different power and electrode gaps on the value of myofibril fragmentation index (MFI) of frozen tilapia fillets. “a–c” letters indicate significant differences (*p* < 0.05). Error bars show standard deviation.

The MFI is a useful indicator of the extent of proteolysis, indicating both the degree of rupturing of the I-bands and the breakage of inter-myofibril linkages [24]. The MFI reflects the integrity of muscle fibers and their skeletal proteins. A higher MFI value corresponds to more severe myofibril breakage and greater meat tenderness. The effects of the electrode gap and power on the degradation of myofibrils are shown in Figure 3. Compared with the 300 W and 900 W samples, the MFI values of the 600 W samples were markedly higher (*p* < 0.05). The MFI in the 10 cm group was significantly higher than that in the 12 cm and 14 cm groups at 300 W (*p* < 0.05), indicating better tenderness at 10 cm, which is consistent with the tenderness reflected by the shear force measurements. In the present study, it is likely that the observed increase or decrease in MFI for the RF-treated samples at different powers for the same electrode gap was related to the chemical and physical changes occurring in the meat due to RF. The MFI in the 300 W-14 cm group was the lowest of all the RF groups, possibly because the slowest electric field deflection and the smallest energy accumulation occurred when the electrode gap was the largest and the power was the highest, resulting in the slow degradation of myofibrils [25].

### 3.3. Temperature Distribution

**Figure 4:**
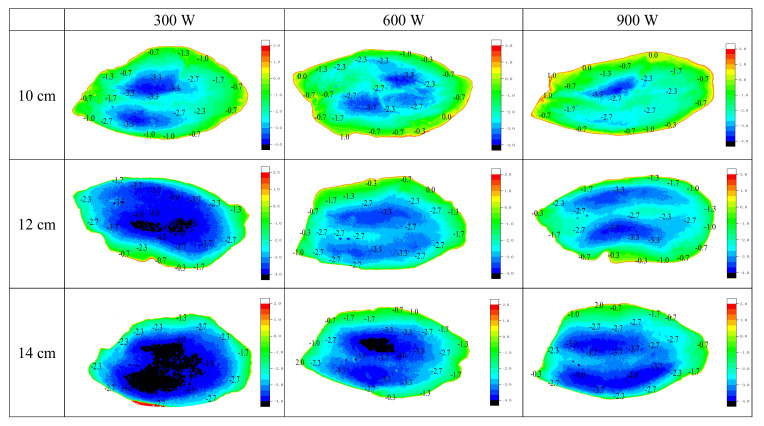
Contour map of the temperature distribution of different RF processing groups.

As shown in Figure 4, the lowest temperature of the sample in the nine RF processing groups was frequently between −3 °C and −4 °C. When the tempering process was terminated, all samples showed a gradual increase in temperature from the center to the edge, with the temperature near the edge of the sample being higher. The part with higher temperature gradually increased as the power increased under the 10 cm electrode gap; the distribution was more uneven, and local overheating was more pronounced in the 900 W samples. This finding is contrary to the trends of springiness, cohesiveness, and resilience under the same conditions. As the temperature increased, the frozen fillet absorbed energy and started to exhibit a phase change. The frozen water at the surface of the fillet began to melt, the water molecules underwent a gradual transition to a less structured state, and a certain amount of water loss resulted; at this point, the internal structure was no longer compact [11].

Alfaifi et al. [26] also reported that, with increasing power, the temperature increased more rapidly in the corners and edges than the center. The edge of the fillet melted faster, and the transition speed of the water molecules was faster because the edge was thinner, and a thermal runaway phenomenon occurred due to an increase in the dielectric loss factor as the temperature increased, which caused a large area of melting at the edge [20]. Farag [27] also indicated that an increase in the power level resulted in a higher average temperature in the fillet within a constant tempering terminal range. The changing trend of the average temperature for different electrode gap groups at the same power was contrary to the trend of increasing hardness, which was consistent with the report of Yang [11]. In addition, the tempering uniformity of the 600 W group was relatively better than that of the other two power groups due to the smaller numbers of overheated and excessively cold areas, and the temperature difference was relatively small, which is consistent with the states of hardness and chewiness under the same conditions. Therefore, this study shows that there is a certain relationship between tempering uniformity and the changes in meat texture during tempering.

### 3.4. Drip Loss and Cooking Loss

The structure of muscle tissue and the degree of shrinkage of myofibrils may be affected by changes in the water loss during tempering, which in turn affects the stability of meat texture [19]. The drip loss increased with increasing electrode gap when the power was 900 W (Table 2). This outcome may be because the tempering time gradually increased as the electrode gap increased, resulting in the release of a large number of oxidases and pro-oxidants, which promoted protein carbonylation reactions and led to the destruction of cell structures, gradually increasing the drip loss. In addition, the drip loss increased with increasing power and a 12 cm electrode gap, which was consistent with the changing trend in shear force under the same conditions. Studies have reported that high power promoted water flow from intra-myofibrillar spaces into the extra-myofibrillar spaces, increasing the water content of extra-myofibrillar spaces, where it was more easily lost and thus increased the drip loss [28]. Therefore, the loss of water from the intra-myofibrillar spaces under this condition destroyed the tissue structure and increased the shear force on the fillets.

Cooking loss includes the loss of moisture and water-soluble ingredients from the flesh. A gradual compression of the meat structure occurs during cooking, as the melting of fat and the denaturation of proteins lead to the release of chemically bound water, which reflects the specific water-holding capacity of the muscles [29]. The results of this study show that cooking loss decreased with the increasing electrode gap for the 600 W samples. The reason may be that the protein solubility was slowly diminished with the increasing electrode gap at a constant voltage, which led to the denatured proteins slowly losing their ability to retain water, with a minor portion released after tempering and another portion released after cooking [30]. These results are consistent with the changing trends of springiness, cohesiveness, and resilience observed under the same conditions.

### 3.5. Moisture Distribution and Migration

**Figure 5:**
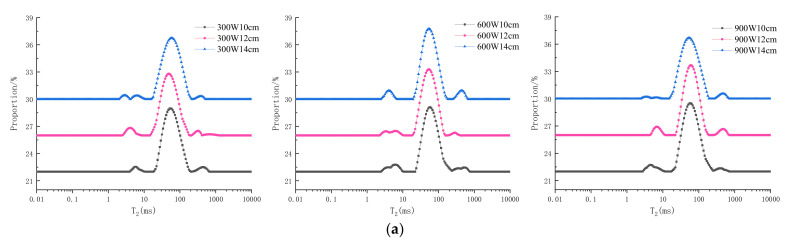

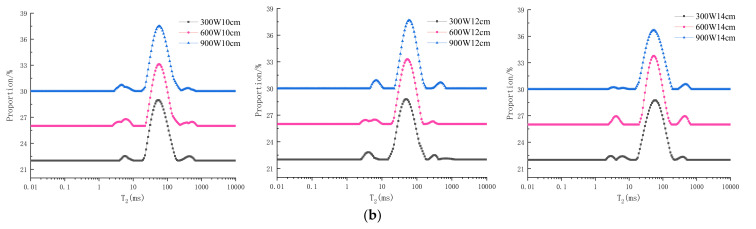
Moisture distribution and migration of frozen tilapia fillets with different RF tempering groups. (**a**) Moisture distribution and migration of frozen tilapia fillets with RF tempering at different electrode gaps (when power is the same). (**b**) Moisture distribution and migration of frozen tilapia fillets with RF tempering at different powers (when electrode gap is the same).

The flow of water destroys cells, causes tissue degradation, and affects the texture of muscles [31]. The water fluidity and the bonding forces between water and meat tissue can be described by the transverse relaxation time (T_2_), as measured by LF-NMR. As shown in Figure 5a,b, the three peaks (T_21_, T_22_, T_23_) were assigned to three key populations of water in muscle: T_21_ (0–10 ms), T_22_ (10–100 ms), and T_23_ (100–1000 ms) represent bound water (bound to macromolecules), immobilized water (in spaces with a high density of myofibrils), and free water (in the myofibril lattice), respectively [30].

In Figure 5a, the bound water relaxation time under the 12 and 14 cm electrode gaps tends to move more to the right than that under the 10 cm gap when the power is 300 W. This suggests that the fraction of water was weakly bound by RF tempering under the 12 and 14 cm electrode gaps, which is consistent with the changing trend in cohesiveness under the same conditions. This result may be related to the protease and microorganisms being more active when the energy conduction was faster due to higher power, causing the decomposition of proteins, which led to an increase in water fluidity. Shao et al. [32] also pointed out that the T_2_ relaxation time of water increased, which indicates that the binding between water and macromolecules was loose and that the water was more fluid.

As shown in Figure 5b, the free water relaxation time at 900 W tended to move more to the right than the 300 W and 600 W groups when the electrode gap was 12 cm. The primary cause for this movement was that the ice crystals gradually melted as the power increased, the free water migrated to the external environment, and the physical adsorption by the meat gradually decreased. The transverse relaxation time (T_2_) typically reflects the bonding force between water and meat tissue, and the area of the peak represents the moisture content [33]. As shown in Table 3, A_21_ gradually decreased as the electrode gap increased for the 600 W group, which was consistent with the changes in springiness and cohesiveness. Other studies have reported similar results; when the binding ability of water was lower, the muscles were looser [34]. The peak area of immobilized water for the 12 cm electrode gap group was higher than that of the other electrode gap groups at 600 W, and the peak area of immobilized water for the 600 W group was higher than that of other power groups under the 12 cm electrode gap, suggesting that the free water refluxed and increased the content of immobilized water under these conditions, and that the sample maintained a satisfactory microstructure, stable conformation, and excellent physicochemical properties [31].

### 3.6. Relationship between Texture and Moisture Index and the Principal Component Analysis (PCA)

**Figure 6:**
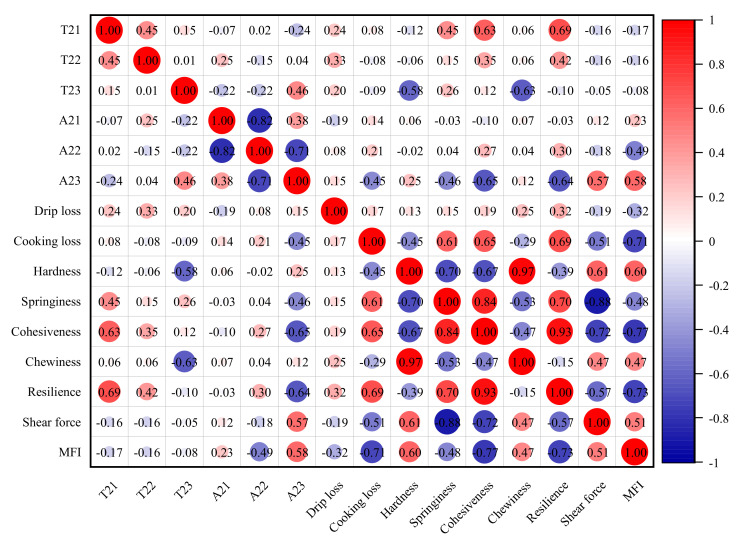
Pearson plot between TPA index and moisture index, shear force, and MFI.

The correlations between moisture characteristics and TPA characteristics, shear force, and MFI for different RF treatments were determined (Figure 6). The shear force and MFI showed a significant positive correlation with A_23_. The springiness, cohesiveness, and resilience showed a negative correlation with A_23_ and a positive correlation with T_21_. The hardness, shear force, and MFI showed a negative correlations with cooking loss. These results indicate that the contents of immobilized water and free water and the cooking loss may have a certain effect on the texture, and that a close correlation exists between textural properties, water migration and water loss during RF tempering, which requires further analysis and confirmation. Some people have reported that the lower hardness values after tempering could be an indicator of the juiciness of the meat and could have an effect on overall consumer acceptability of the meat [13]. However, in this study, the drip loss was weakly correlated with the texture index, and this result may be related to the short tempering time.

**Figure 7:**
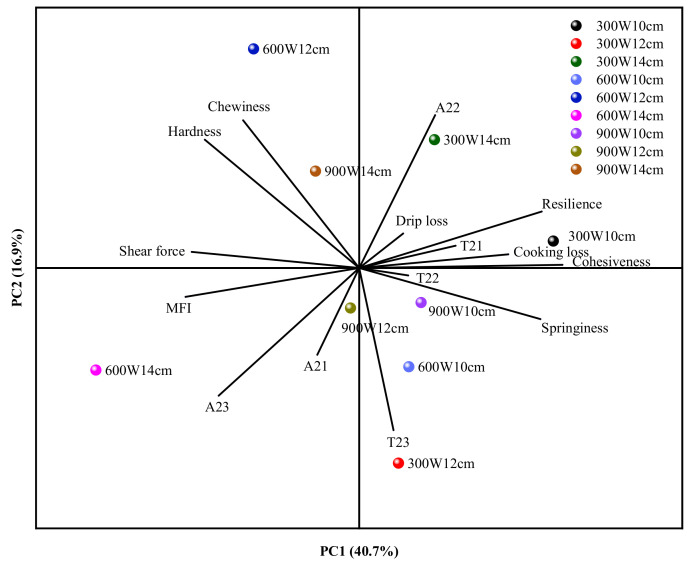
Analysis of principal components (PCA) of the texture index (hardness, cohesiveness, chewiness, resilience, springiness), moisture index (NMR index (T_21_, T_22_, T_23_, A_21_, A_22_, A_23_), drip loss and cooking loss), shear force and MFI (myofibril fragmentation index) of all samples.

The PCA biplot shown in Figure 7 simultaneously represents the observations and variables in the new space, where the first two principal components (PCs) explain 57.60% of the total variation (PC1 and PC2 were 40.70 and 16.90%, respectively). The first major component (PC1), which represents 40.70% of the variability, most often describes resilience, springiness, cooking loss, and cohesiveness variables, with a negative association with chewiness, hardness, shear force, MFI, and A_23_ variables, as shown in Figure 6. In the first component, only samples in the 300 W-10 cm RF group were associated with higher resilience, springiness and cohesiveness, indicating that the better springiness and cohesiveness gain associated with the 300 W-10 cm RF treatment resulted in a compact myofibrillar structure, which is consistent with the results shown in Table 1. PC2 was defined by chewiness and hardness, which were negatively correlated with A_23_ and T_23_, indicating that the content of free water caused a change in the texture of the tempered fillets. For component 2 (positive side of graphic), only the 14 cm group with 900 W was associated with greater hardness, chewiness, A_22_ and cohesiveness, which indicates that the hardness and chewiness increased as the electrode gap increased to 14 cm for the 900 W group, and the immobilized water can be prevented from becoming free water, as shown in Figure 6.

This study investigated the texture of fillets after RF tempering and the associated mechanisms that caused their differences. Previous studies had observed that RF tempering can affect the texture and other qualities of products; however, most of these were focused on exploring the effects on color, water loss, and fat oxidation, and less attention was paid to the texture [11,13,17,18,19]. Bedane et al. [13] reported that a higher hardness of chicken after RF tempering was achieved by increasing the electrode gap. In this study, we also proved that the hardness of fillets gradually increased with the increasing electrode gap at 900 W. Zhang et al. [18] found that there was no significant difference (*p* > 0.05) in hardness, chewiness, springiness, or resilience in RF-tempered fillets with alterations in the power and electrode gap. However, our results indicate a decreasing trend in springiness, cohesiveness, and resilience as the electrode gap increased, which is consistent with Bedane et al.’s result [13]. Compared to previous research, the variations in temperature distribution, water holding capacity, and moisture distribution after tempering were not only observed in our study, and we also assessed the mechanisms for difference in texture after tempering. Finally, the pivotal factors that led to the difference in texture after tempering were confirmed by the correlation analysis and principal component analysis.

## 4. Conclusions

This study focused on exploring the texture change mechanism of RF-tempered frozen food products under different RF conditions by analyzing the factors that affect the texture. The results show that the power or the electrode gap can affect the texture of tempered fillets. At 600 W, the springiness decreased from 0.84 mm to 0.79 mm, the cohesiveness decreased from 0.64 mm to 0.57 mm, and the resilience decreased from 0.33 mm to 0.25 mm as the electrode gap was increased. The shear force was increased from 15.18 N to 16.98 N by increasing the power, while the electrode gap remained at 12 cm. Besides this, good tempering uniformity can improve the hardness and chewiness of tempered fillets. The free water content, cooking loss and migration of bound water to immobilized water may be the main factors in the texture change observed after RF tempering. However, deeper and more exact mechanisms of texture change during RF tempering still need to be determined.

## Figures and Tables

**Table 1:** Effects of power and electrode gap on the texture profile analysis (TPA) results and shear force of frozen tilapia fillets.

Electrode Gap (cm)	Power (W)	Hardness (g)	Springiness (mm)	Cohesiveness (mm)	Chewiness (mJ)	Resilience (mm)	Shear Force (N)
	300	162.01 ± 8.15 ^Cb^	0.85 ± 0.01 ^Aa^	0.67 ± 0.01 ^Aa^	91.35 ± 7.08 ^Bb^	0.34 ± 0.01 ^Aa^	11.77 ± 0.42 ^Bb^
10	600	216.22 ± 16.83 ^Ab^	0.84 ± 0.01 ^Aa^	0.64 ± 0.03 ^Aa^	116.22 ± 11.14 ^Ab^	0.33 ± 0.03 ^Aa^	13.36 ± 0.53 ^Ab^
	900	188.01 ± 6.15 ^Bc^	0.83 ± 0.00 ^Aa^	0.64 ± 0.01 ^Aa^	100.92 ± 1.43 ^ABb^	0.32 ± 0.01 ^Aa^	13.27 ± 0.15 ^Ab^
	300	137.66 ± 8.14 ^Cc^	0.82 ± 0.06 ^Ac^	0.63 ± 0.00 ^Ab^	71.41 ± 8.83 ^Cc^	0.30 ± 0.01 ^Aa^	15.18 ± 1.30 ^Aa^
12	600	293.81 ± 30.72 ^Aa^	0.80 ± 0.00 ^Ab^	0.61 ± 0.01 ^Aab^	142.36 ± 15.85 ^Aa^	0.31 ± 0.03 ^Aa^	16.45 ± 1.68 ^Aa^
	900	221.38 ± 13.45 ^Bb^	0.81 ± 0.03 ^Aa^	0.64 ± 0.03 ^Aa^	114.68 ± 3.26 ^Ba^	0.33 ± 0.04 ^Aa^	16.98 ± 0.60 ^Aa^
	300	218.08 ± 17.47 ^Ba^	0.81 ± 0.03 ^Ab^	0.64 ± 0.03 ^Aab^	113.20 ± 9.44 ^Aa^	0.33 ± 0.03 ^Aa^	14.06 ± 1.12 ^Ba^
14	600	263.95 ± 13.58 ^Aa^	0.79 ± 0.01 ^Ab^	0.57 ± 0.01 ^Ab^	119.35 ± 3.62 ^Ab^	0.25 ± 0.02 ^Ab^	16.51 ± 0.43 ^Aa^
	900	247.02 ± 12.92 ^ABa^	0.81 ± 0.05 ^Aa^	0.61 ± 0.05 ^Aa^	122.06 ± 8.23 ^Aa^	0.30 ± 0.05 ^Aa^	15.76 ± 1.41 ^ABa^

A–C Mean values in the same column with the same electrode gap followed by different letters are significantly different according to Duncan’s multiple range test (*p* < 0.05). a–c Mean values in the same column with the same power followed by different letters are significantly different according to Duncan’s multiple range test (*p* < 0.05).

**Table 2:** Effects of power and electrode gap on drip loss and cooking loss of frozen tilapia fillets.

Electrode Gap (cm)	Power (W)	Drip Loss (%)	Cooking Loss (%)
10	300	0.43 ± 0.06 ^BY^	15.71 ± 0.31 ^AY^
	600	0.84 ± 0.06 ^AX^	17.84 ± 0.99 ^AX^
	900	0.31 ± 0.09 ^BY^	15.90 ± 0.05 ^AY^
12	300	0.16 ± 0.01 ^BY^	17.34 ± 1.03 ^AX^
	600	0.17 ± 0.00 ^CY^	15.94 ± 0.37 ^AXY^
	900	0.57 ± 0.10 ^ABX^	14.20 ± 0.07 ^CY^
14	300	0.85 ± 0.19 ^AX^	16.55 ± 0.44 ^AX^
	600	0.38 ± 0.01 ^BY^	11.08 ± 0.49 ^BY^
	900	0.76 ± 0.08 ^AXY^	15.44 ± 0.03 ^BX^

A–C Mean values in the same column with the same power followed by different letters are significantly different according to Duncan’s multiple range test (*p* < 0.05). X, Y Mean values in the same column with the same electrode gap followed by different letters are significantly different according to Duncan’s multiple range test (*p* < 0.05).

**Table 3:** Peak areas of moisture content of frozen tilapia fillets tempered by different RF groups.

Electrode Gap (cm)	Power (W)	A_21_	A_22_	A_23_
10	300	2.73 ± 0.26 ^Bb^	94.05 ± 0.90 ^Aa^	3.22 ± 0.65 ^Aa^
	600	6.98 ± 1.13 ^Aa^	89.17 ± 2.93 ^Aa^	3.85 ± 1.80 ^Aa^
	900	5.25 ± 1.54 ^ABa^	92.63 ± 1.42 ^Aa^	2.12 ± 0.12 ^Aa^
12	300	5.27 ± 1.03 ^Aa^	91.67 ± 0.73 ^Ab^	3.06 ± 1.75 ^Aa^
	600	5.52 ± 0.70 ^Aa^	93.21 ± 0.25 ^Aa^	1.27 ± 0.45 ^Aa^
	900	5.15 ± 1.04 ^Aa^	91.18 ± 1.74 ^Aa^	3.67 ± 0.70 ^Aa^
14	300	4.11 ± 0.46 ^Aab^	94.36 ± 0.36 ^Aa^	1.53 ± 0.10 ^Aa^
	600	5.13 ± 0.13 ^Aa^	89.80 ± 1.02 ^Ba^	5.07 ± 1.15 ^Aa^
	900	1.67 ± 0.69 ^Ba^	95.22 ± 1.41 ^Aa^	3.11 ± 2.11 ^Aa^

A–C Mean values in the same column with the same electrode gap followed by different letters are significantly different according to Duncan’s multiple range test (*p* < 0.05). a, b Mean values in the same column with the same power followed by different letters are significantly different according to Duncan’s multiple range test (*p* < 0.05).

## Data Availability

The datasets generated for this study are available on request to the corresponding author.
